# Nuclear Magnetic Resonance Spectroscopy for In Situ Monitoring of Porous Materials Formation under Hydrothermal Conditions

**DOI:** 10.3390/ma11081416

**Published:** 2018-08-12

**Authors:** Mohamed Haouas

**Affiliations:** Institut Lavoisier de Versailles, CNRS, UVSQ, Université Paris-Saclay, 45 av. des Etats-Unis, 78330 Versailles, France; mohamed.haouas@uvsq.fr; Tel.: +33-139-254-254

**Keywords:** NMR spectroscopy, zeolites, zeotypes, metal-organic frameworks, aluminophosphates, aluminosilicates, synthesis precursors, nucleation and growth, crystallization, mechanism

## Abstract

The employment of nuclear magnetic resonance (NMR) spectroscopy for studying crystalline porous materials formation is reviewed in the context of the development of in situ methodologies for the observation of the real synthesis medium, with the aim of unraveling the nucleation and growth processes mechanism. Both liquid and solid state NMR techniques are considered to probe the local environment at molecular level of the precursor species either soluble in the liquid phase or present in the reactive gel. Because the mass transport between the liquid and solid components of the heterogeneous system plays a key role in the synthesis course, the two methods provide unique insights and are complementary. Recent technological advances for hydrothermal conditions NMR are detailed and their applications to zeolite and related materials crystallization are illustrated. Achievements in the field are exemplified with some representative studies of relevance to zeolites, aluminophosphate zeotypes, and metal-organic frameworks.

## 1. Introduction

Crystalline microporous solids historically include zeolites and zeotype materials mostly metallophosphates, and recently hybrid organic-inorganic coordination polymers also called metal organic frameworks (MOFs), has joined this family of compounds [1]. They are extensively used in various fields such as catalysis, separation, ion exchange, and gas storage [2,3,4,5,6]. Understanding their properties imposes a deep knowledge about their chemistry and structure. The three-dimensional structures of these classes of materials are often described as an assembly of well-defined molecular building blocks traditionally called secondary building units or SBUs [7,8]. The shape, size, and the symmetry of these SBUs define the final topology of the framework structure and the porous system. Solvent and host molecules are also often found entrapped within their cages and channels interacting strongly with the framework and are suspected to play key role to ensure the structural cohesion [9,10]. Although they can be removed through postsynthesis procedures, the extra-framework species are usually present in as-made materials. The template effect is now recognized as an important phenomenon occurring during formation of these solids [11,12,13,14,15]. The course of their synthesis depends not only on hydrolysis-polycondensation processes but also governed by the supramolecular chemistry [16,17].

Unraveling crystallization mechanism of microporous compounds is of great interest in engineering tunable synthesis of materials with specific properties for target applications. Synthesis of microporous is a complex process because of the heterogeneity of the synthesis medium, hardly reproducible, composed of gel or mixture of insoluble solids and solution or in the best cases sols. Moreover, it is a multiparameter synthesis depending on various physical and chemical factors like temperature, ageing time, pressure, pH, molar composition, concentration, solvent, etc. Nevertheless, extensive studies have been carried out, and some general concepts about crystallization mechanisms of these materials have been proposed including solution-mediated nucleation-growth processes and solid-solid transformation models [8,16,18,19,20,21,22]. Nucleation and growth from soluble prefabricated SBUs or related molecular structures is among the most accepted mechanisms [8,23,24], although more elementary mechanisms like growing from monomers and small oligomers have also been suggested in the literature [25,26]. By opposition, evolution of amorphous nanoparticles developing ordered structures internally constitutes an alternative model to liquid transport crystallization [27,28]. All these models, and others, need validation from theory and experiments. Combination of various techniques to explore wide temporal and spatial domain scales is a required approach to refine as much as possible the current crystallization models of the different synthesis systems. 

To verify such postulated mechanisms, kinetic investigation of the crystallization process represents one of the most effective experimental approaches [29,30,31]. These methods became more asserted through in situ approach, enabling sufficient number of experimental data required for precise kinetics and thermodynamics analyses [32]. Since reactive and intermediate species formed during the crystallization are fragile and difficult to separate from their native synthesis medium without changing its properties, in situ methodologies present much more advantages by comparison to ex situ procedures [33,34]. Furthermore, in situ techniques offer more reliable information of crystallization events on real time. The majority of in situ studies of following the microporous crystallization process over time have focused on the formation kinetics of the Bragg reflections. Understanding the assembly of crystallites from amorphous precursors at the molecular level at the early stages of crystallization still remains a great challenge because of the lack of long-range order. To directly observe chemical reactions and formation steps within the solvent prior to solid precipitation local scale spectroscopic methods are however needed. Compared to solid-state analyses, only a few studies have been carried out on the early stages of microporous solids formation in solution in real synthesis conditions. To get a complete picture, both liquid and solid parts of the system have to be analyzed in situ through specific techniques. Indeed, various in situ studies have been reported covering both liquid and solid state aspects, often separately, including Infrared and Raman spectroscopies [19,35,36], X-ray absorption spectroscopy [8,37], X-ray/neutron diffraction (XRD) [29,36,38,39], small- and Wide-angle X-ray scattering (SAXS/WAXS) [40,41], atomic force microscopy [33,42], electrochemical impedance spectroscopy [43], and NMR/magic angle spinning (MAS) NMR [8,28,36,38]. 

Among other spectroscopic methods, NMR has been frequently used in microporous formation studies, since it gives the possibilities of gaining detailed information about speciation in both solid and solution phases [18,44]. This technique provides structural and dynamic insights with distinct spatial and temporal resolution. NMR spectroscopy allows access to unique information on the short-range (0.15–0.3 nm length scale) and medium-range (0.3–1 nm) structures and on motions over wide timescale range (from femtosecond to second). Furthermore, this spectroscopy is element specific and inherently quantitative. NMR spectroscopy is ideally suited to the study of microporous materials since they contain numerous active NMR nuclei like ^29^Si, ^27^Al, ^31^P, ^19^F, ^1^H, and ^13^C in both framework and extra-framework parts. Multinuclear approach is capable of providing distinct information on local structures of organic/inorganic species, and their interaction, simultaneously [45,46,47]. In situ NMR has been progressively developed to investigate the real synthesis conditions of microporous solids, under hydrothermal conditions. It starts with the pioneering works of Taulelle et al. in the mid-1990s adapted for liquid-state NMR apparatus [48,49,50], and only recently real in situ high temperature high pressure MAS NMR technology has been achieved at the Pacific Northwest National Laboratory (PNNL) [28,51,52]. 

I will survey herein the recent technological and methodological developments in the field of in situ NMR of hydrothermal synthesis medium together with past results. Within this context, I will expose an overview of main achievements in mechanistic studies of microporous materials crystallization through representative examples of zeolites, aluminophosphates, and aluminum carboxylate type MOFs.

## 2. Some Experimental Aspects of NMR under Hydrothermal Conditions 

### 2.1. NMR Cells and Devices for High Temperature and High Pressure

**Figure 1:**
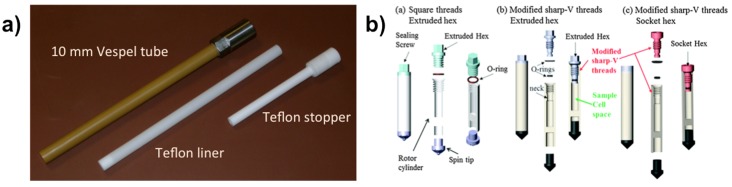
NMR cells for hydrothermal conditions: (**a**) 10 mm tube for liquid state NMR; (**b**) representative designs of high temperature and high pressure MAS rotors. Adapted from [51,54].

Zeolites and related compounds including MOFs are usually synthesized under mild hydrothermal conditions (*T* < 250 °C, *P* < 40 bars) [16]. In situ NMR measurements of synthesis media under real conditions require specific equipment. Although conventional NMR with modern spectrometers can reach high temperatures up to 150 °C routinely (when equipped with an air-cooling system), sample holders having to withstand combined high temperature and high pressure is a real issue. High-pressure NMR tubes exist commercially but are made in glass, which could be a source of contamination when exposed to the corrosive synthesis media, especially at high temperatures. Taulelle et al., who first used in 1995 homemade NMR tubes for hydrothermal conditions [53], succeeded to measure corrosive media (HF containing solutions at extreme pH) at temperature exceeding 210 °C under autogenesis pressure [54]. For solid-state NMR, rotors have to be sealed tightly under high pressure during MAS. This requires not only mechanical resistance against temperature and pressure but also high rotation speed stability, which represents a real challenge. Recently, Hu et al. from PNNL in Richland (USA), also reported homemade devises for solid-state NMR allowing measurement up to 250 °C and 100 bars under 4 kHz MAS [51]. These devices for liquid and solid-state NMR are shown in Figure 1 acting as real hydrothermal cells where the synthesis is performed inside the NMR magnet. 

The hydrothermal cell for liquid NMR (Figure 1a) is made from a Vespel (or Torlon) 10 mm tube, protected with a Teflon liner to avoid any contact with the reaction medium. Sealing is ensured with an elongated Teflon screw to reduce as maximum as possible sample volume. The hydrothermal MAS rotors (Figure 1b) are made from zirconia cylinders with sizes ranging from 9.5 to 3.2 mm. The rotor can be sealed with a screw cap fitting one or two O-rings, and at its opposite side a spin tip is fixed in a separate compartment. This design is capable of sealing heterogenous mixed solid-liquid samples under extreme experimental conditions.

Such specific setups for NMR measurements under combined high temperature and high pressure conditions are realized with special diamagnetic resistant materials to avoid any perturbation and interference with both the external high magnetic field (*B*_0_) and the radio-frequency field (*B*_1_). These devices are thus designed with metallic character-free components using high performance polymers (Vespel, Torlon, Teflon, etc.), ceramics (zirconia), and resins (epoxy glue, etc.). These achievements represent an important technological breakthrough to perform in situ NMR measurements under harsh hydrothermal conditions. Nevertheless, NMR studies of hydrothermal media are still rather scarce probably because they are no without risk. 

### 2.2. Measuring pH In Situ by NMR Method

Proton activity, usually expressed as pH, is one of the most critical parameters in hydrolysis/polycondensation chemistry of oxides in aqueous solution. It is obvious that it plays crucial role in the synthesis of microporous compounds since speciation in solution is directly dependent on pH. However, measuring the pH in the synthesis conditions at high temperature and pressure is technically a difficult task. Usually this parameter is measured at room temperature before or/and after the hydrothermal synthesis, but such values could not reflect the real reaction pH. An NMR method for pH probing of hydrothermal solutions has thus been developed [48] using the NMR tubes for autogeneous pressures at high temperature shown in Figure 1a.

**Figure 2:**
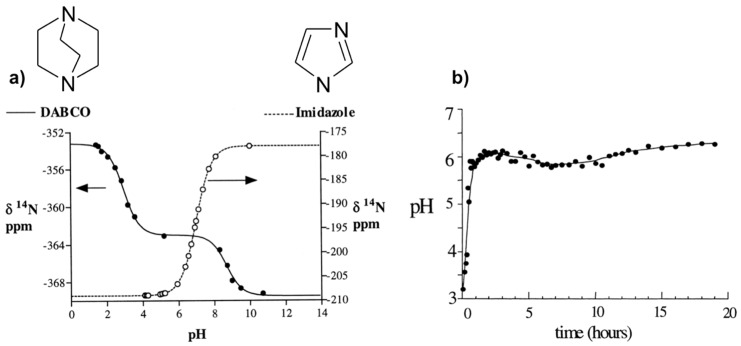
NMR method to measure the pH in situ: (**a**) ^14^N chemical shifts of Im and DABCO versus pH; (**b**) in situ pH evolution with time during the hydrothermal synthesis of AlPO_4_-CJ2 at 150 °C calculated from calibration curves of NMR parameter as a function of pH shown in (a). Adapted from [48].

The method consists of using internal molecular probes as pH indicators through the dependence of its chemical shifts with pH. Two amines, imidazole (Im), and 1,4-diazabicyclo[2,2,2]octane (DABCO), have been selected on the base of their complementary *pKa* values to cover a wide pH range (about 9 pH units). Indeed, ^14^N chemical shifts are sensitive to pH change close to the *pKa* values where the population of protonated species varies significantly. To establish the relationships between the observed chemical shifts (*δ_obs_*) and the pH, calibration curves depending of the amine have to be performed first. From such calibration, expression of pH can be derived (Equation (1)) on the base of fast chemical exchange regime on the NMR time scale between the different protonation states (BH^+^/B) of the amine B at pH around the corresponding *pKa_BH_^+^_/B_*.

(1) $$\mathrm{pH}=pKa_{BH^{+}/B}+\mathrm{Log}\frac{\left(\delta_{obs}-\delta_{BH^{+}}\right)}{\left(\delta_{B}-\delta_{obs}\right)}$$

where *δ_BH_^+^* and *δ_B_* are chemical shifts of each protonated/deprotonated species. These characteristic parameters were determined for Im with one protonation state (*pKa_ImH_^+^_/Im_*, *δ_ImH_^+^*, and *δ_Im_*) and DABCO with two protonation states (*pKa_DABCOH2_*^2^*^+^_/DABCOH_^+^*, *δ_DABCOH2_*^2^*^+^*, *δ_DABCOH_^+^*, *pKa_DABCOH_^+^_/DABCO_*, *δ_DABCOH_^+^*, and *δ_DABCO_*), from which the calibration curves are calculated (Figure 2a). These parameters are also temperature dependent and have to be calibrated for each desired temperature [48].

This method has been applied to follow the pH evolution during the hydrothermal synthesis of the microporous AlPO_4_-CJ2 at 150 °C (Figure 2b). Results indicate a rapid pH increase from approximately 3.5 to 6 during the first hour and then a fairly constant pH value at 6 along the synthesis period. The jump of the pH at the early stage of the synthesis can be explained by a rapid dissolution of an initially formed amorphous phase and the pH stabilization around 6 reveals pH conditions close to neutral since *pK_W_* = 11.5 at 150 °C. This is important information with respect to Al speciation in solution known to be pH dependent.

### 2.3. Quantification by NMR at Variable High Temperature

Among information needed to investigate mechanisms of crystallization process from heterogenous medium, knowledge about the amount of soluble species is crucial not only to understand solution speciation but also solubility and supersaturation effects. NMR spectroscopy is a quantitative technique in the sense that the intensity of the NMR response is directly proportional to the quantity of detected nuclei. Nevertheless, on increasing the temperature of samples, considerable NMR signal loss occurs as a consequence of Curie’s law. Dielectric constant and conductivity effects on signal loss are reflected in the evolution of the quality factor (*Q*) of the probe. A calibration method for signal loss correction based on relative variations of the *Q* factor of the radiofrequency (rf) circuit is thus necessary for efficient spin counting. The relations between acquisitions, transmission and reception, and experimental NMR amplitudes had been studied in details in the context of NMR under hydrothermal conditions [55].

**Figure 3:**
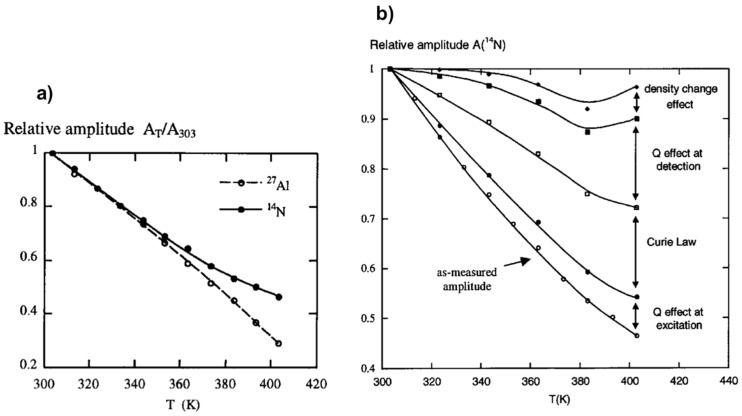
NMR response dependence with temperature: (**a**) Temperature effect on ^27^Al and ^14^N NMR amplitude losses; (**b**) experimental ^14^N NMR amplitude as a function of temperature and successive corrections from effects of *Q*, Currie law, and density change. Adapted from [55].

Figure 3a shows the as-measured amplitudes, *A*, of the ^27^Al and ^14^N NMR signals of an aluminum nitrate solution monitored as a function of temperature. A substantial NMR amplitude loss of about 70% is observed on heating from 303 to 403 K in the case of ^27^Al and about 55% in the case of ^14^N. Several factors have been identified contributing to these signal losses as follows:

(i) Currie Law effect: At a given external magnetic strength *B*_0_ the magnetization *M*_0_ and the magnetic susceptibility *χ*_0_ varies as 1/*T*:

(2) $$M_{0}=\chi_{0}B_{0};\;\chi_{0}\;\propto \;\frac{1}{T};\;\Rightarrow \frac{A_{T}}{A_{303}}\;\propto \;\frac{303}{T}$$

(ii) Sample density effect: The amplitude *A* should be directly proportional to liquid density ρ which is temperature dependent:

(3) $$\frac{A_{T}}{A_{303}}\;\propto \;\frac{\rho_{T}}{\rho_{303}}$$

(iii) *Q* factor effect at excitation: The amplitude *A* is dependent on the pulse angle *θ* and in turn to the pulse length *t_p_* with respect to the 90° pulse length *t*_90_:

(4) $$\frac{A_{T}}{A_{303}}\;\propto \;\frac{\sin(\theta_{T})}{\sin(\theta_{303})}\;\Rightarrow \;\frac{A_{T}}{A_{303}}\;\propto \;\frac{\sin(\frac{90t_{p}}{t_{90}^{T}})}{\sin(\frac{90t_{p}}{t_{90}^{303}})}$$

(iv) *Q* factor effect at detection: The amplitude *A* is also a function of *Q* factor of the probe head that can be measured independently using a network analyzer or through the values of the measured 90° pulse length *t*_90_ at a given temperature:

(5) $$\frac{A_{T}}{A_{303}}\;\propto \;\sqrt{\frac{Q_{T}}{Q_{303}}}\Rightarrow \;\frac{A_{T}}{A_{303}}\;\propto \;\sqrt{\frac{t_{90}^{T}}{t_{90}^{303}}}$$

Correction from all these effects allows recovering the NMR amplitude loss as it has experimentally been verified on the example of ^14^N NMR of aluminum nitrate solution (Figure 3b). The two major contributors to the NMR signal loss upon the temperature variation have found to be the Curie law effect and probehead quality factor change. This latter is mainly due to electrical conductivity and dielectric constant changes of the sample. Preliminary studies of specific calibration of some variables like ρ(*T*) and *t*_90_(*T*) are required. This method appears very useful under specific conditions for which internal calibration is difficult. It allows the quantification of hydrothermal NMR at variable temperature experiments considering the effects of not only temperature, but also concentration of solutes and pH which affect significantly the conductivity and dielectric properties of the medium.

## 3. Examples of In Situ NMR Studies on Crystallization of Microporous Materials

### 3.1. Zeolites

#### 3.1.1. First In Situ MAS NMR Study

The first report on the use of solid-state NMR to monitor in situ the evolution of order during the conversion of amorphous intermediate gels to crystalline zeolites dates to 1996. Carr and coworkers studied the formation of zeolite A acquiring ^29^Si and ^27^Al MAS spectra at 65 °C over a period of ca. 5 h [56]. They used conventional MAS equipment and standard rotors since this zeolitic system offers the possibility of quick crystallization at moderate temperature from gel composition in the molar range 1 Al_2_O_3_:2 SiO_2_:2–4 Na_2_O:40–160 H_2_O. The MAS rate was also moderate at ca. 2 kHz.

**Figure 4:**
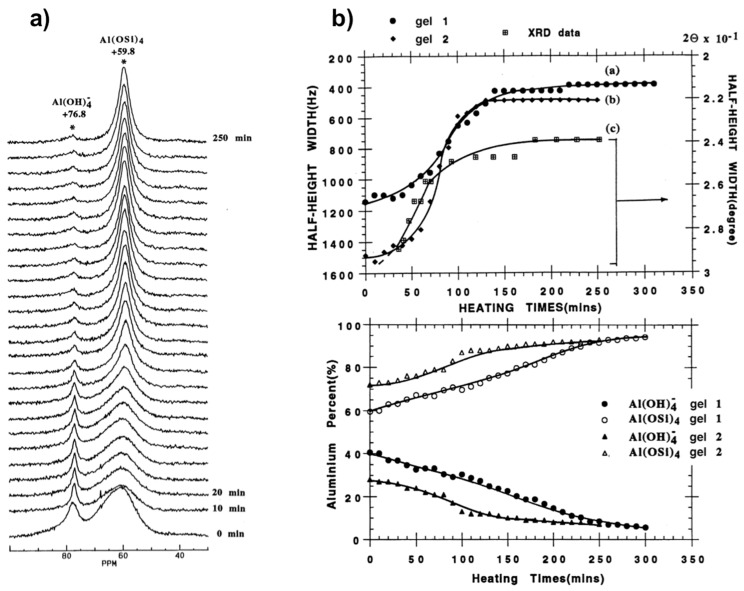
Monitoring the zeolite A synthesis from two intermediate sodium aluminosilicate gels (gel 1:1 Al_2_O_3_:2 SiO_2_:4.3 Na_2_O:160 H_2_O; gel 2:1 Al_2_O_3_:2 SiO_2_:2.4 Na_2_O:42 H_2_O): (**a**) In situ ^27^Al MAS NMR spectra from gel 2 at 65 °C. (**b**) Top: plots showing synthesis progress as measured from line narrowing of 59 ppm ^27^Al line and 2*θ* 29.9° Bragg peak; Bottom: curves of Al% in the liquid phase (close symbols) and the gel phase (open symbols) from quantitative ^27^Al NMR vs heating time. Adapted from [56].

Figure 4 shows the results obtained from in situ ^27^Al MAS NMR spectra of silicoaluminate zeolite A synthesis from gel at 65 °C [56]. These spectra (Figure 4a) allowed monitoring the growth of the crystalline phase and the species occurring in solution and the gel phases. A rapid increase in crystallinity can be revealed from decreasing half-height width of the peak at 59 ppm associated to framework Al(OSi)_4_ species as a function of the crystallization time. Meanwhile, the peak intensity of [Al(OH)_4_]^−^, representing the soluble species, decreases gradually indicating that these latter participate directly in the framework development of zeolite A during the crystallization process. Thus ordering of the local environment of aluminum tetrahedra with formation of long-range Al(OSi)_4_ can be monitored in situ by ^27^Al NMR spectroscopy in conjunction with XRD (Figure 4b).

A study as a function of gel composition showed that water affects significantly kinetics of crystallization process. More concentrated gel promotes crystallization and the transformation rate increases suggesting that more nuclei should be formed during the induction/nucleation period. There is however no indication from the ^29^Si MAS NMR measurements of specific solution-state species, such as secondary building units, most probably due to their existence as a broad structural and conformational distribution that could not be resolved at the NMR time scale.

#### 3.1.2. Liquid State In Situ NMR Study

**Figure 5:**
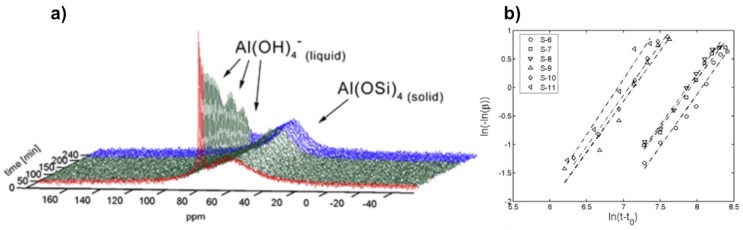
Kinetics analysis of zeolite A crystallization: (**a**) Typical in situ ^27^Al NMR spectra during the course of zeolite A synthesis monitored by liquid-state NMR technique; (**b**) examples of linear Sharp-Hancock plots obtained from ^27^Al NMR kinetic curves describing crystallization of zeolite A for different molar compositions. Adapted from [30].

The same system, i.e., zeolite A, has also been subjected to in situ NMR study using conventional liquid state NMR. Miladinovic et al. used 10 mm Quartz tubes to study suspension (dilute gel) precursors for zeolite A at relatively mild temperatures not exceeding 80 °C [30,57]. With this method, they identified signatures for species in both liquid and solid phases that they can monitor simultaneously during the synthesis course (Figure 5). The species in liquid phase are characterized by a narrow resonance as a result of rapid tumbling in solution, whereas the solid-state components appear broad due to reduced mobility and conformational distribution. 

Similar to the previous in situ MAS study, crystallization profiles from these ^27^Al NMR spectra, where an example is shown in Figure 5a, can be derived either from the increase of the broad line at 59 ppm or the decrease of the narrow line at 79 ppm. The simultaneous changes in intensity and shape of both NMR lines are indicative of depletion of [Al(OH)_4_]^−^ ions from the liquid phase and the building up of a Al(OSi)_4_ tetrahedral network at the surface of zeolite crystal particles [30]. Both alkalinity and dilution effects have been investigated on various gels with wide molar composition range. A particular interest has been attracted to quantitative analysis.

By using the Sharp-Hancock kinetic model (Equation (6)), crystallization parameters including Avrami’s exponent *n* and rate constant *k*, can be extracted for a given system (Figure 5b). The crystallization extent *α* derived from ^27^Al NMR data can be expressed as a function of time *t* following Equation (6):

(6) $$\ln\left[-\ln\left(1-\alpha \right)\right]=n\;\ln\left(t-t_{0}\right)+n\;\ln k$$

where *t*_0_ stands for induction time. The crystallization exponent *n* also known as Avrami’s exponent provides useful insight about crystallization mechanism. Indeed, low values of *n* (typically 0.5 < *n* < 1.5) are usually observed for diffusion-controlled mechanism, while higher values (typically 2.0 < *n* < 3.5) are indicative of phase boundary growth process [30]. It has been found that syntheses performed at lower alkalinity conditions provide high values of *n* ranging from 1.9 and 3.3, consistent with Avrami-Erofe’ev nucleation and crystal growth model. On the other hand, the almost dominant presence of a diffusion mechanism has been verified for syntheses conducted at higher alkalinity conditions with values for *n* gathered around 1.5.

### 3.2. Aluminophosphate Zeotypes

Aluminophosphate molecular sieves have been considered as model compounds to study crystallization of zeolitic materials offering quite simpler chemistry compared to the complex gel chemistry of alkaline aluminosilicate systems. Férey pioneered the development of molecular building block concept for rational synthesis of open framework solids [1,58,59]. Such molecular “Lego” approach for the bottom-up synthesis of complex architectures needs the identification of reactive SBU species in order to control the course of the synthesis, and thus to validate the concept. For this means, Férey and coworkers developed in situ methodologies to probe metallophosphate microporous synthesis media including XRD, EXAFS, and NMR [8,29,60]. For the latter, oxyfluorinated alumonphosphate AlPO_4_-CJ2, (NH_4_)_0.88_(H_3_O)_0.12_AlPO_4_(OH_0.33_F_0.67_), has been chosen as the model compound for the multinuclear NMR approach [61]. 

#### 3.2.1. Identifying the Primary Building Units (PBUs)

**Figure 6:**
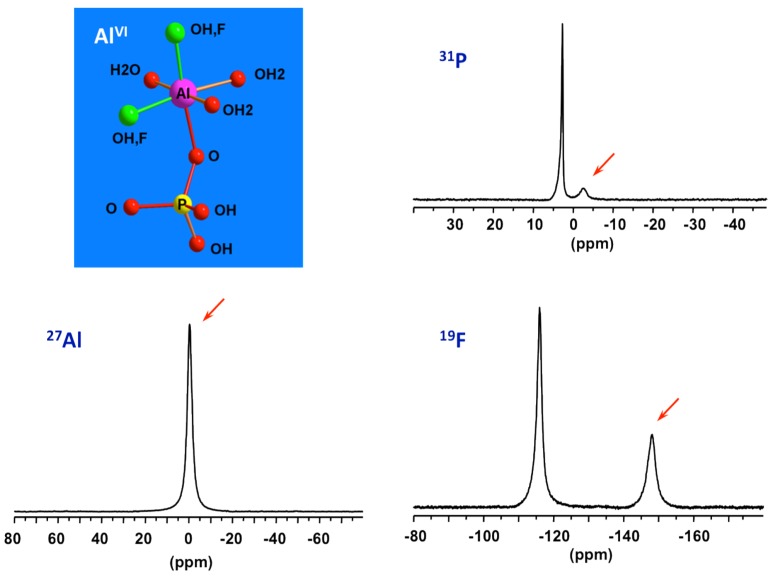
^27^Al, ^31^P, and ^19^F NMR spectra of solution part of the starting precursor for AlPO_4_-CJ2 prior to heating showing signatures (red arrows) of PBUs, octahedral mixed fluoroaluminophosphates.

By combining data from ^31^P, ^27^Al and ^19^F NMR of solution part of the initial precursor of AlPO_4_-CJ2, the nature of the species constituting the reactive medium prior to heating has been identified [49]. Specific signatures for Al-OP and Al-F bonds can be recognized from the ^31^P and ^19^F NMR spectra, respectively. This precursor has been found to be very simple, mainly composed of mixed fluoroaluminophosphate octahedral complexes accompanied by freely solvated fluoride and phosphate anions (Figure 6). These complexes should be thermodynamically stable and poorly reactive at ambient conditions since they are formed spontaneously upon mixing the starting reagents. Such elementary species should therefore represent the primary building units or PBUs from which more complex structures could be derived. PBUs include the elementary building units, such as the tetrahedral phosphate anions and octahedral aluminum cations but also the very simple fluoroaluminophosphate complexes.

#### 3.2.2. Tracking the Prenucleation Building Units (PNBUs)

**Figure 7:**
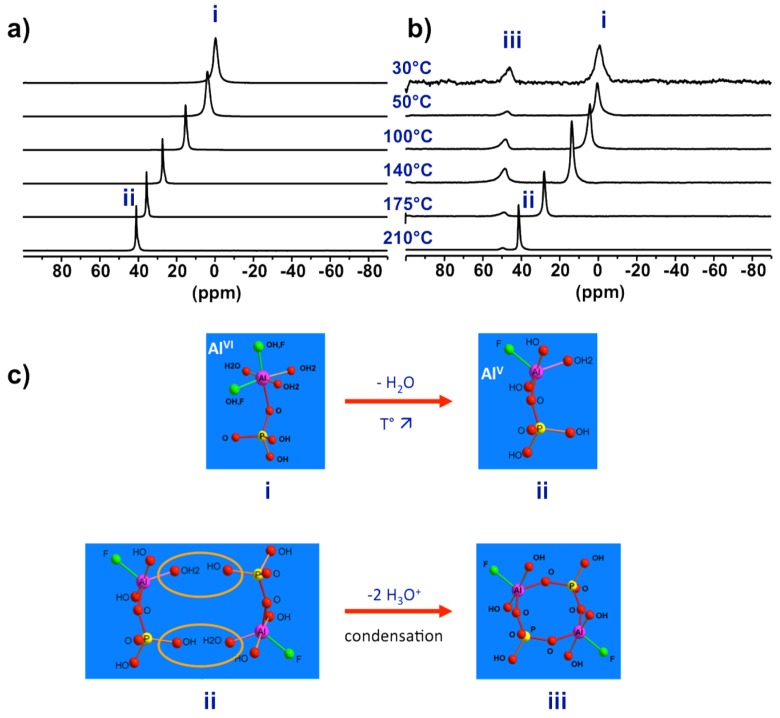
Evidence of reactive species for AlPO_4_-CJ2 crystallization, the prenucleation building unit (PNBU): Variable temperature ^27^Al NMR spectra of (**a**) the liquid part of the initial precursor showing the change in coordination number of Al in the PBU and of (**b**) the heterogeneous solid-liquid complete precursor showing the formation of new species (PNBU) with temperature-independent reduced coordination number; (**c**) proposed sequential pathway of PNBU formation from starting PBU.

To investigate the real synthesis medium high temperature NMR under hydrothermal conditions is required. Using devices shown in Figure 1a, ^27^Al NMR was successively applied on precursor synthesis of AlPO_4_-CJ2 [50]. The main results are shown in Figure 7. When increasing progressively temperature up to 210 °C the ^27^Al NMR line of PBU shifts continuously from 0 to 40 ppm indicating a coordination change from octahedral at room temperature to penta-coordinated Al at synthesis temperature (Figure 7a). In this experiment, only the liquid part of the synthesis mixture was considered after separation of the insoluble solid component by filtration. In a second experiment taking into account the overall heterogeneous system without phase separation (Figure 7b), new resonances appeared at high temperature at ca. 50 ppm. Interestingly, lowering the temperature back to room conditions led to the shift of the PBU line from 40 to 0 ppm, as observed previously in the first experiment on solid-separated liquid, but did not affect the position of the new resonance which remain at 50 ppm even at room temperature.

From these results, one can conclude that the starting primary complexes undergo coordination change from hexa-coordinated Al, species (i) in Figure 7, at room temperature to more reactive penta-coordinated Al species (ii) at synthesis temperature. Without the presence of the initial solid part of the precursor, these species (ii) are not reactive enough to partially transform into the third new species (iii) at 50 ppm. This supposes supersaturation conditions needed for such transformation. The NMR signature of species (iii) is temperature-independent meaning these species are metastable over a short period of time where its coordination state is maintained through equilibrium with growing solid particles. Such observations typically apply to nucleating reactive species at work. 

These results represent experimental evidence of the so-called prenucleation building units or PNBUs, which differ structurally from the SBU for the construction of the solid as it will be discussed in next section. A sequential pathway from initial hexa-coordinated PBU species (i) to the PNBU species (iii) passing by the intermediate reduced coordination number species (ii) is proposed in Figure 7c. The PNBUs consist of cyclic dimerized form of the penta-coordinated PBUs. The reduction in coordination number by rising the temperature facilitates condensation reactions.

#### 3.2.3. Structural Relationship between PNBUs and SBUs

**Figure 8:**
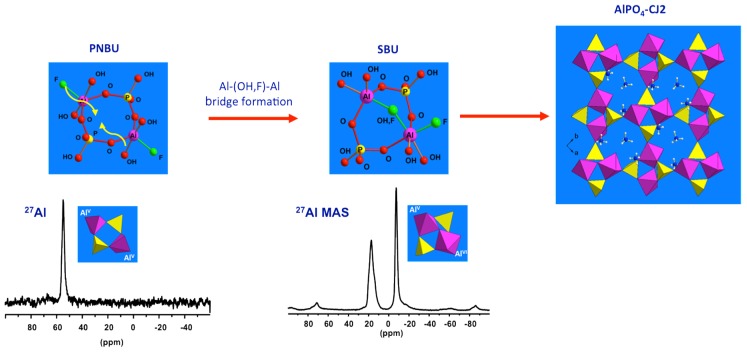
Proposed evolution from PNBU to SBU to extended solid by successive internal and inter-unit condensations. The structural relationship between the PNBU in synthesis solution and SBU in final solid is emphasized through comparison of their ^27^Al NMR signatures.

Now the prenucleating species (PNBU) are identified experimentally, their comparison with the AlPO_4_-CJ2 SBU can reveal some insights about the crystallization process for this compound. The PNBU is a tetrameric unit composed of two aluminum and two phosphate units linked to each other by alternation. The two Al centers adopt penta-coordinated geometry according to the unique resonance observed in solution at 55 ppm in a dissolution experiment at 210 °C (Figure 8), or at 50 ppm in the synthesis medium during the crystallization (Figure 7b). The SBU is structurally comparable showing a tetrameric unit but the two aluminums are bridged and also differ from their coordination number where one is octahedrally coordinated and the second is present in penta-coordinated environment. This is well-illustrated in the MAS spectrum of the solid exhibiting the two distinct resonances.

It is clear that the PNBU differs from the SBU, but they are structurally related. A simple junction between the two aluminum centers in the PNBU by linking them through one terminal function (OH or F) is sufficient to create the SBU structure. This means by a simple conformational rearrangement, the PNBU can be transformed into the AlPO_4_-CJ2 SBU. This would occur during the condensation of these PNBUs under structural constrain imposed by the minimization of the network energy to create the growing surface. Also, as an evidence of that, the bridged position between the two Al atoms in the SBU was found to be distributed, i.e., partially occupied by either a hydroxide or a fluoride suggesting that such particular site would be created in a later stage during the AlPO_4_-CJ2 network development [62]. In this study case of AlPO_4_-CJ2, although SBU has not been identified in growth solution, structurally related PNBU has been clearly evidenced thanks to in situ NMR.

#### 3.2.4. Recent MAS In Situ Investigation on AlPO_4_-5

Since the first in situ measurements by the MAS NMR technique exposed in Section 3.1.1, limited studies have been achieved certainly due to the technical challenge with respect to high pressure at high temperature conditions. Most of the in situ NMR investigations are conducted under static condition that suffers from spectral resolution associated with the solid part or at moderate temperature below 100 °C in an in situ MAS setup that restricts the study to only a few systems [35]. Thanks to recent availability of high temperature-high pressure MAS rotors, presented in Figure 1b, in situ multinuclear MAS NMR investigations of the crystallization process of AlPO_4_-5 molecular sieve have been carried out [28]. Especially, the roles of water during the crystallization were demonstrated by highly sensitive in situ ^1^H MAS NMR.

**Figure 9:**
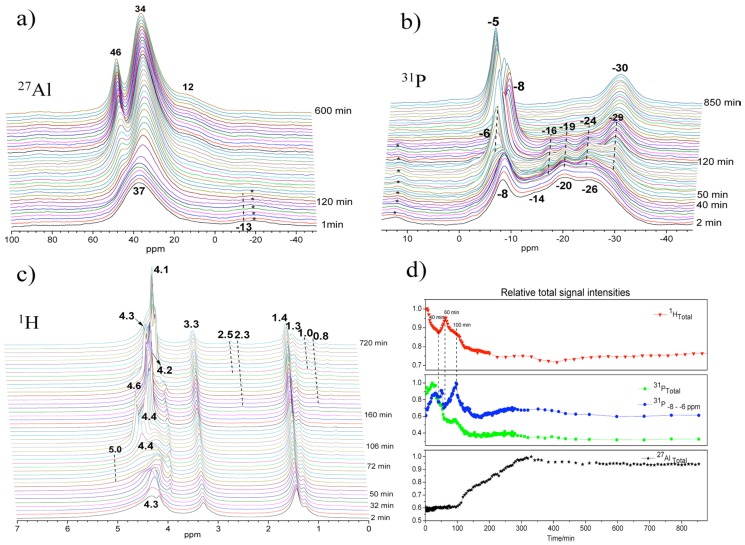
Time on stream in situ MAS NMR spectra and their relative signal intensities of synthesis gel crystallized at 150 °C: (**a**) ^27^Al, (**b**) ^31^P, (**c**) ^1^H MAS NMR, and (**d**) normalized signal intensities. Adaptive from [28].

^27^Al, ^31^P, and ^1^H MAS NMR spectra of synthesis gel for AlPO_4_-5 at 150 °C maintained over a period of 14 h are shown in Figure 9. After about 100 min, the peak of the four-coordinated aluminum (Figure 9a) became narrower and gradually shifted from 37 to 34 ppm, attributed to the framework Al of AlPO_4_-5. Solution species were also detected and are represented by the 46 ppm peak appearing at extended reaction time. In ^31^P spectra (Figure 9b), the peak of terminal phosphate (PO_3_(OAl)) near −8 ppm became broad as a new shoulder peak rose at −6 ppm at about 50 min. Correspondingly, relative signal intensities of these ^31^P lines increased significantly (Figure 9d), implying that the breakage of Al-O-P bond occurs in the amorphous gel as more terminal phosphates are formed. With crystallization proceeding, the chemical shift oscillating combined with fluctuated change of the signal intensity of the −6 and −8 ppm peaks seems to indicate repeated hydrolysis and condensation reaction, producing and consuming terminal phosphate functions during the whole synthesis process.

Thus, specific water should catalyze the structure rearrangement via repeated hydrolysis and condensation reaction. Indeed, in ^1^H MAS NMR (Figure 9c), significant changes were demonstrated near 4–5 ppm belonging to hydroxyl groups, protonated amine, and water that are all involved in an exchange process. The activated water with sharp peak features at 4.3 ppm at 30 min dispersed in different environments with well distinguishable peaks near 4–5 ppm at 50–70 min. Such water should play an important role during the hydrolysis of Al-O-P bonds. The significantly fluctuated change of ^1^H signal intensity during the initial 100 min seems to indicate continuous adjustment of local structures of amorphous gel accompanied by excluding or consuming excess water, phosphate, and aluminum species.

### 3.3. Aluminum Carboxylate MOFs

MOFs, like zeolites are usually synthesized by the hydrothermal technique, but they also are obtained many often solvothermally when an organic solvent is employed to solubilize, as much as possible, poorly soluble organic linkers. In such a case, the synthesis conditions are usually milder compared to the hydrothermal synthesis when these solvents present lower vapor pressure and higher boiling point than those of water. Because ^27^Al is a relatively high-sensitivity nucleus with 100% natural abundance, most in situ NMR studies have focused on the synthesis of Al-based MOFs, where both ^1^H and ^27^Al NMR can be used to probe the formation process. Two main contributions in the field have been published so far on MIL-type Al carboxylate systems (MIL stands for Material of Institute Lavoisier) [63,64].

#### 3.3.1. Identifying PNBUs in Growth Solution of Al-Trimesate Based MOFs

Aluminum trimesate (1,3,5-benzene tricarboxylate, or btc) is typical system within which several compounds can be formed by varying the reaction conditions and synthesis parameters. Indeed, three phases with completely different three-dimensional crystal structures appear in this system by varying the pH and reaction time. The common precursor is a mixture of Al source and trimesate ester where the molar ratio and pH are adjusted depending on the nature of the final product. The complex structure of MIL-96 [65], consists of corrugated chains of octahedral Al forming hexagonal 18-membered ring tunnels, at the center of which are fixed μ_3_-oxo-centered trinuclear Al_3_ clusters. Such trinuclear units are also found in the mesoporous MIL-100 and represents the unique SBU for this phase [66]. The third compound, MIL-110 [67], is built up from completely different an original SBU based on Al_8_ octamer. 

At short reaction times (<5 h), increasing the pH successively leads to MIL-100 in a very narrow range of pH (0.5 < pH < 0.75), MIL-96 (0.75 < pH < 3.25), and MIL-110 (pH > 3.25). Above 60 h of reaction, the repartition has completely changed: MIL-100 has disappeared, and MIL-110, which was formed in 4 h at pH 3.5, exists now only at very low pH (<0.5), with the MIL-96 domain being almost unchanged. This surprising behavior has been investigated by the combination of X-ray powder diffraction and in situ NMR method [64].

**Figure 10:**
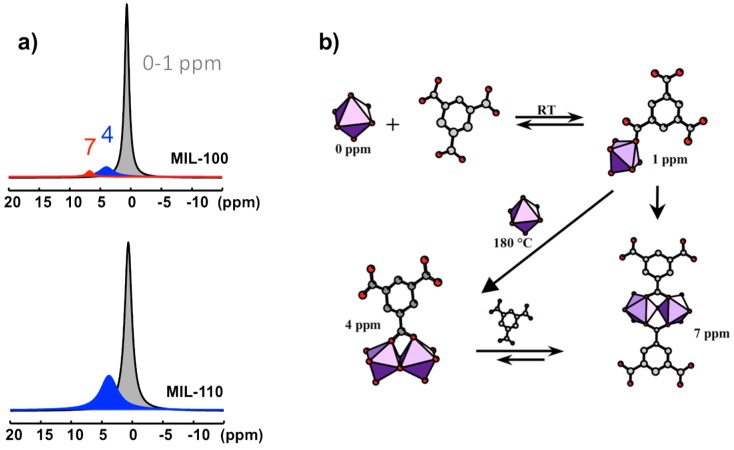
Al-based species observed in the synthesis solutions of aluminum trimesate MOF type compounds, MIL-96, MIL-100, and MIL-110: (**a**) Typical ^27^Al NMR spectra of synthesis medium for MIL-100 and MIL-110 recorded at 180 °C; (**b**) chemical pathways of the starting PBUs leading to the PNBUs characterized by the 4 and 7 ppm ^27^Al signals.

Four ^27^Al NMR signals can be distinguished at 0, ~1, ~4, and ~7 ppm (Figure 10a). They correspond to four distinct Al-based species in octahedral coordination. Their identification is based on comparison between NMR observation in solution and the nature of the XRD solid product along the syntheses course. The signal at 0 ppm is observed in all the solutions. It is assigned to the uncomplexed cation Al(H_2_O)_6_^3+^. The resonance at 1 ppm appears during the increase of the temperature, from room temperature to 180 °C. Its presence is correlated with the presence of btc in solution, and therefore assigned to the primary complex Al(H_2_O)_5_(H_2_btc)^2+^. This labile complex undergoes fast chemical exchange with Al(H_2_O)_6_^3+^, and these two species represent the PBUs. The signals at 4 and 7 ppm are visible only at synthesis temperature 180 °C. The signal at 4 ppm correlates with the MIL-110 formation, while the appearance of the second signal (7 ppm) coincides with the formation of MIL-96 and, to a lesser extent, the formation of MIL-100. MIL-110 is based on a unique octameric Al_8_ unit, whereas MIL-96 and MIL-100 share a common trimeric Al_3_ unit, Al_3_(μ_3_-O)(H_2_O)_2_(OH)(btc)_2_. As the 7 ppm signal appears always after the 4 ppm signal, the two corresponding species should be structurally related. Therefore, the substructure Al_2_(μ_2_-O)(H_2_O)_2_(btc)_2_^2−^ (corner-sharing bi-octahedral motif) would be more likely related to the 7 ppm signal, knowing that MIL-110 presents another kind of dimer Al_2_(μ_2_-O)_2_(H_2_O)_2_(btc)^−^ (edge-sharing bi-octahedral motif), which would be related to the 4 ppm signal. On this basis, the resonances at 4 and 7 ppm are assigned to the dimer complexes Al_2_(μ_2_-OH)_2_(H_2_O)_6_(H_2_btc)^3+^ and Al_2_(μ_2_-OH)(H_2_O)_6_(H_2_btc)_2_^3+^, respectively (Figure 10b).

**Figure 11:**
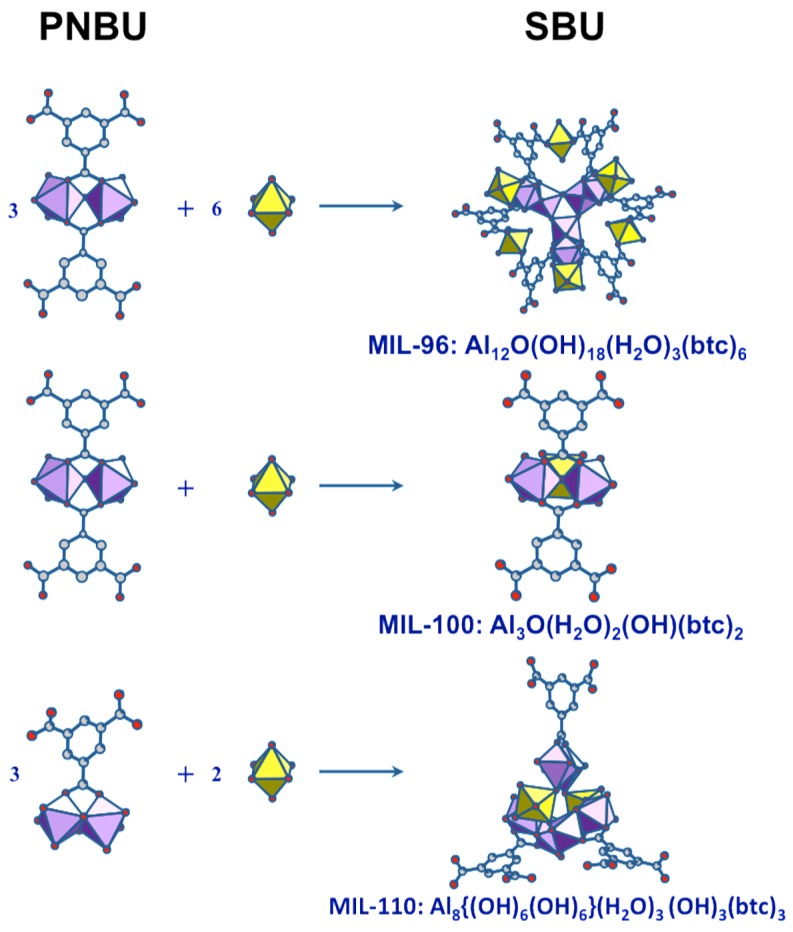
Structural relationship between the PNBUs observed in synthesis solutions and the SBUs of the aluminum trimesates MIL-96, MIL-100, and MIL-110.

According to the Férey SBU concept [58], the species present in solution at the moment of crystallization are directly related to the building units of the crystal. Since the SBUs of the three products (MIL-96, MIL-100, and MIL-110) are very different and complex, the two resonances observed in synthesis solutions should correspond to simpler but related substructures. These PNBUs can lead to the final corresponding SBUs simply by adding monomers (Figure 11).

#### 3.3.2. The Role of *N*,*N*-Dimethylformamide Solvent on the Synthesis of NH_2_-MIL-101

In another study of the MIL series, Gascon et al. used in situ MAS NMR to elucidate the role of *N*,*N*-dimethylformamide (DMF) in promoting NH_2_-MIL-101(Al) formation [63]. The NMR measurements were in essence a liquid-state experiments, but in order to detect large chemical structures in confined space that are normally beyond the typical limits of detection in solution NMR, the synthesis precursors were rotated under magic angle at a sample rotation rate of 1.1 kHz. For this purpose a Bruker 7 mm MAS WVT (wide variable temperature) probe head was used. To contain the pressure buildup at the synthesis temperature, i.e., 130 °C, specially designed home-constructed PEEK (polyether ether ketone) inserts sealable with screwed caps were used inside the standard zirconia 7 mm MAS rotors. Nevertheless, the generated pressure should be moderate since the experiment temperatures did not exceed the boiling point of DMF (153 °C) used as solvent.

**Figure 12:**
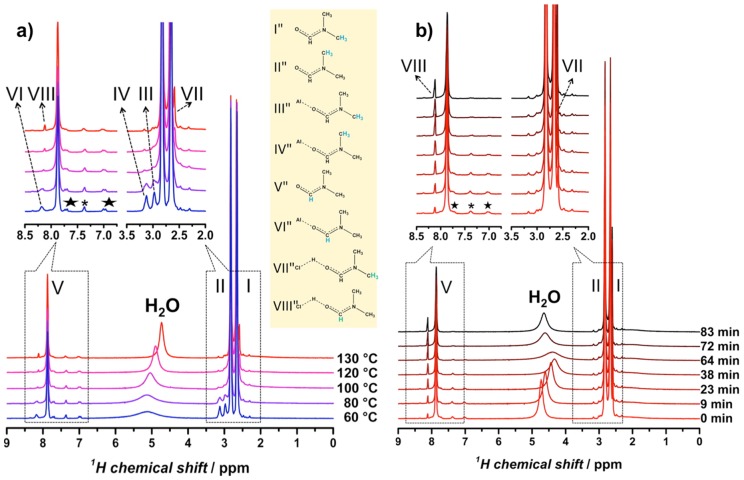
In situ ^1^H MAS NMR spectra of NH_2_-MIL-101(Al): (**a**) Evolution of the spectra as a function of temperature and (**b**) evolution of the spectra as a function of time at fixed temperature, 130 °C. Adapted from [63].

From earlier XRD experiments [68], it was determined that either NH_2_-MIL-101(Al) or NH_2_-MIL-53(Al) could form from a common NH_2_-MOF-235(Al) intermediate, and that solvent effects (DMF versus H_2_O) played a critical role in determining the final product. Building on this work, Gascon et al. identified a number of protonated complexes with DMF occurring at high temperature in their ^1^H NMR spectra (Figure 12a) [63]. They showed that ^1^H NMR peaks assigned to H−Cl···DMF grew as a function of time (Figure 12b) and were concurrent with a downfield-shift and broadening of the ^1^H NMR signal for water. These observations led the authors to conclude that DMF serves as a molecular promoter for a water dissociation reaction (Equation (7)) that transforms water-coordinated NH_2_-MOF-235(Al) into hydroxy-coordinated NH_2_-MIL-101(Al).

(7) $${\mathrm{Cl}}^{-}+H_{2}O+\mathrm{DMF}\;\to {\;\mathrm{OH}}^{-}+H-\mathrm{Cl}\cdots \mathrm{DMF}$$

Moreover, these results correlate well with the consumption of ^27^Al nuclei in solution.

## 4. Conclusions

In situ NMR techniques yield invaluable information at the molecular scale on processes occurring in the transformation of growth solutions and amorphous precursors for zeolitic matters. With the recent technical progresses and technological advances in the design of specific high-temperature and high-pressure NMR setups, the in situ NMR approach under hydrothermal conditions is now state-of-the-art, although such studies remain still scarce. Various NMR cells and devices including tubes, rotors, and inserts have been developed acting as real hydrothermal reactors for both liquid and solid state NMR. Specific NMR methodologies for hydrothermal conditions, such as high-temperature NMR quantification and in situ NMR pH-metry have been presented. We can expect that these new methodological developments will expand applications in many fields when access to reaction medium by conventional techniques is difficult.

The hydrothermal and solvothermal chemistries are typically hardly accessible by classical analytical tools. The equilibriums and species distributions are different from ambient conditions and in situ investigations are needed for determining chemical composition of synthetic medium and its reactivity. Although elementary building units, i.e., PBUs, can be identified at room and moderate temperatures, the reactive species and PNBUs have been found to occur exclusively at synthesis temperature often with short live-times. The roles of solvent and ‘activated water’ in hydrolysis-condensation are demonstrated. The change in coordination and evolution of pH with temperature affect drastically the stability and thus the reactivity of these PBUs. Kinetics data are also valuable insights that can be obtained only at real conditions.

Despite significant progress in understanding zeolite and MOF syntheses, much work remains to be accomplished since no universal mechanism can be validated for all systems yet.

## References

[1] Loiseau T., Ferey G. (2007). Crystalline oxyfluorinated open-framework compounds: Silicates, metal phosphates, metal fluorides and metal-organic frameworks (MOF). J. Fluor. Chem..

[2] Alhamami M., Doan H., Cheng C.H. (2014). A Review on Breathing Behaviors of Metal-Organic-Frameworks (MOFs) for Gas Adsorption. Materials.

[3] Ennaert T., Van Aelst J., Dijkmans J., De Clercq R., Schutyser W., Dusselier M., Verboekend D., Sels B.F. (2016). Potential and challenges of zeolite chemistry in the catalytic conversion of biomass. Chem. Soc. Rev..

[4] Gkaniatsou E., Sicard C., Ricoux R., Mahy J.P., Steunou N., Serre C. (2017). Metal-organic frameworks: A novel host platform for enzymatic catalysis and detection. Mater. Horiz..

[5] Janiak C., Henninger S.K. (2013). Porous Coordination Polymers as Novel Sorption Materials for Heat Transformation Processes. Chimia.

[6] Morris R.E., Wheatley P.S. (2008). Gas storage in nanoporous materials. Angew. Chem. Int. Ed..

[7] Cantu D.C., McGrail B.P., Glezakou V.A. (2014). Formation Mechanism of the Secondary Building Unit in a Chromium Terephthalate Metal-Organic Framework. Chem. Mater..

[8] Ferey G., Haouas M., Loiseau T., Taulelle F. (2014). Nanoporous Solids: How Do They Form? An In Situ Approach. Chem. Mater..

[9] Cooper E.R., Andrews C.D., Wheatley P.S., Webb P.B., Wormald P., Morris R.E. (2004). Ionic liquids and eutectic mixtures as solvent and template in synthesis of zeolite analogues. Nature.

[10] Ferey G. (2001). Microporous solids: From organically templated inorganic skeletons to hybrid frameworks … ecumenism in chemistry. Chem. Mater..

[11] Catlow C.R.A., Coombes D.S., Pereira J.C.G. (1998). Computer modeling of nucleation, growth, and templating in hydrothermal synthesis. Chem. Mater..

[12] Gies H., Marler B. (1992). The structure-controlling role of organic templates for the synthesis of porosils in the system SiO_2_/template/H_2_O. Zeolites.

[13] Ilyushin G.D., Blatov V.A. (2015). Modeling of self-organization processes in crystal-forming systems: Templated precursor nanoclusters T48 and the self-assembly of crystal structures of 15-crown-5, Na-FAU, 18-crown-6, Na-EMT, and Ca,Ba-TSC zeolites. Russ. J. Inorg. Chem..

[14] Jin L., Auerbach S.M., Monson P.A. (2010). Modeling Nanoparticle Formation during Early Stages of Zeolite Growth: A Low-Coordination Lattice Model of Template Penetration. J. Phys. Chem. C.

[15] Verstraelen T., Szyja B.M., Lesthaeghe D., Declerck R., Van Speybroeck V., Waroquier M., Jansen A.P.J., Aerts A., Follens L.R.A., Martens J.A. (2009). Multi-level Modeling of Silica-Template Interactions During Initial Stages of Zeolite Synthesis. Top. Catal..

[16] Cundy C.S., Cox P.A. (2005). The hydrothermal synthesis of zeolites: Precursors, intermediates and reaction mechanism. Microporous Mesoporous Mater..

[17] Wiebcke M. (1991). Structural links between zeolite-type and clathrate hydrate-type materials. J. Chem. Soc. Chem. Commun..

[18] Epping J.D., Chmelka B.F. (2006). Nucleation and growth of zeolites and inorganic mesoporous solids: Molecular insights from magnetic resonance spectroscopy. Curr. Opin. Colloid Interface Sci..

[19] Fan F.T., Feng Z.C., Li C. (2010). UV Raman spectroscopic study on the synthesis mechanism and assembly of molecular sieves. Chem. Soc. Rev..

[20] Mazaj M., Kaucic V., Logar N.Z. (2016). Chemistry of Metal-organic Frameworks Monitored by Advanced X-ray Diffraction and Scattering Techniques. Acta Chim. Slov..

[21] Reinsch H., Stock N. (2017). Synthesis of MOFs: A personal view on rationalisation, application and exploration. Dalton Trans..

[22] Walton R.I., Norquist A., Smith R.I., O’Hare D. (2003). Recent results from the in situ study of hydrothermal crystallisations using time-resolved X-ray and neutron diffraction methods. Faraday Discuss..

[23] Embrechts H., Kriesten M., Hoffmann K., Peukert W., Hartmann M., Distaso M. (2018). Elucidation of the Formation Mechanism of Metal-Organic Frameworks via in-Situ Raman and FTIR Spectroscopy under Solvothermal Conditions. J. Phys. Chem. C.

[24] Wragg D.S., Morris R.E. (2001). Synthesis and structure determination from an extremely small single crystal of a new layered gallium phosphate. J. Phys. Chem. Solids.

[25] Belton D.J., Deschaume O., Perry C.C. (2012). An overview of the fundamentals of the chemistry of silica with relevance to biosilicification and technological advances. FEBS J..

[26] Chien S.C., Auerbach S.M., Monson P.A. (2015). Modeling the Self-Assembly of Silica-Templated Nanoparticles in the Initial Stages of Zeolite Formation. Langmuir.

[27] Rao C.N.R., Dan M., Behera J.N. (2005). Chemical design of materials: A case study of inorganic open-framework materials. Pure Appl. Chem..

[28] Zhao Z.C., Xu S.C., Hu M.Y., Bao X.H., Hu J.Z. (2016). In Situ High Temperature High Pressure MAS NMR Study on the Crystallization of AlPO_4_-5. J. Phys. Chem. C.

[29] Loiseau T., Walton R.I., Francis R.J., O’Hare D., Ferey G. (2000). Open-framework fluorinated gallium and aluminium phosphates: An in situ study of the hydrothermal synthesis by X-ray diffraction using synchrotron radiation. J. Fluor. Chem..

[30] Miladinovic Z.P., Zakrzewska J., Kovacevic B.T., Miladinovic J.M. (2014). In situ Al-27 NMR kinetic investigation of zeolite A crystallization. Microporous Mesoporous Mater..

[31] Heidenreich N., Rutt U., Koppen M., Inge A.K., Beier S., Dippel A.C., Suren R., Stock N. (2017). A multi-purpose reaction cell for the investigation of reactions under solvothermal conditions. Rev. Sci. Instrum..

[32] Cheetham A.K., Kieslich G., Yeung H.H.M. (2018). Thermodynamic and Kinetic Effects in the Crystallization of Metal-Organic Frameworks. Acc. Chem. Res..

[33] Kumar M., Choudhary M.K., Rimer J.D. (2018). Transient modes of zeolite surface growth from 3D gel-like islands to 2D single layers. Nat. Commun..

[34] Van Vleet M.J., Weng T.T., Li X.Y., Schmidt J.R. (2018). In Situ, Time-Resolved, and Mechanistic Studies of Metal-Organic Framework Nucleation and Growth. Chem. Rev..

[35] Aerts A., Kirschhock C.E.A., Martens J.A. (2010). Methods for in situ spectroscopic probing of the synthesis of a zeolite. Chem. Soc. Rev..

[36] Depla A., Lesthaeghe D., van Erp T.S., Aerts A., Houthoofd K., Fan F.T., Li C., Van Speybroeck V., Waroquier M., Kirschhock C.E.A. (2011). Si-29 NMR and UV-Raman Investigation of Initial Oligomerization Reaction Pathways in Acid-Catalyzed Silica Sol-Gel Chemistry. J. Phys. Chem. C.

[37] Souleiman M., Cambon O., Haidoux A., Haines J., Levelut C., Ranieri V., Hazemann J.L. (2012). Study of Ga^3+^-Induced Hydrothermal Crystallization of an alpha-Quartz Type Ga1-xFexPO_4_ Single Crystal by in Situ X-ray Absorption Spectroscopy (XAS). Inorg. Chem..

[38] Anderson S.L., Gladysiak A., Boyd P.G., Ireland C.P., Mieville P., Tiana D., Vlaisavljevich B., Schouwink P., van Beek W., Gagnon K.J. (2017). Formation pathways of metal-organic frameworks proceeding through partial dissolution of the metastable phase. CrystEngComm.

[39] Xia F., O’Neill B., Ngothai Y., Peak J., Tenailleau C., Etschmann B., Qian G.J., Brugger J., Studer A., Olsen S. (2010). A thermosyphon-driven hydrothermal flow-through cell for in situ and time-resolved neutron diffraction studies. J. Appl. Crystallogr..

[40] O’Brien M.G., Beale A.M., Weckhuysen B.M. (2010). The role of synchrotron radiation in examining the self-assembly of crystalline nanoporous framework materials: From zeolites and aluminophosphates to metal organic hybrids. Chem. Soc. Rev..

[41] Zhao X.M., Liu R.G., Zhang H., Shang Y.S., Song Y., Liu C., Wang T., Gong Y.J., Li Z.H. (2017). Structure evolution of aluminosilicate sol and its structure-directing effect on the synthesis of NaY zeolite. J. Appl. Crystallogr..

[42] Wagia R., Strashnov I., Anderson M.W., Attfield M.P. (2016). Determination of the Preassembled Nucleating Units That Are Critical for the Crystal Growth of the Metal-Organic Framework CdIF-4. Angew. Chem. Int. Ed..

[43] Brabants G., Lieben S., Breynaert E., Reichel E.K., Taulelle F., Martens J.A., Jakoby B., Kirschhock C.E.A. (2016). Monitoring early zeolite formation via in situ electrochemical impedance spectroscopy. Chem. Commun..

[44] Li S.H., Deng F., Webb G.A. (2013). Annual Reports on NMR Spectroscopy.

[45] Brunner E. (1995). Solid-state NMR—A powerful tool for the investigation of surface hydroxyl-groups in zeolites and their interaction with adsorbed probe molecules. J. Mol. Struct..

[46] Koller H., Weiss M., Chan J.C.C. (2012). Solid State NMR.

[47] Zhang L., Ren Y.H., Yue B., He H.Y. (2012). Recent development in in situ NMR study on heterogeneous catalysis: Mechanisms of light alkane functionalisation. Chem. Commun..

[48] Gerardin C., In M., Allouche L., Haouas M., Taulelle F. (1999). In situ pH probing of hydrothermal solutions by NMR. Chem. Mater..

[49] Haouas M., Gerardin C., Taulelle F., Estournes C., Loiseau T., Ferey G. (1998). In situ NMR study of hydrothermal synthesis of a template-mediated microporous aluminophosphate material: AlPO_4_-CJ2. J. Chim. Phys. Chim. Biol..

[50] Taulelle F., Haouas M., Gerardin C., Estournes C., Loiseau T., Ferey G. (1999). NMR of microporous compounds—From in situ reactions to solid paving. Colloid Surf. A Physicochem. Eng. Asp..

[51] Hu J.Z., Hu M.Y., Zhao Z.C., Xu S.C., Vjunov A., Shi H., Camaioni D.M., Peden C.H.F., Lercher J.A. (2015). Sealed rotors for in situ high temperature high pressure MAS NMR. Chem. Commun..

[52] Xu S.C., Zhao Z.C., Hu M.Y., Han X.W., Hu J.Z., Bao X.H. (2016). Investigation of water assisted phase transformation process from AlPO_4_-5 to AlPO_4_-tridymite. Microporous Mesoporous Mater..

[53] In-gerardin C., In M., Taulelle F. (1995). In-situ NMR measurements under hydrothermal conditions—Study of the formation of polymeric Al hydrolysis species. J. Chim. Phys. Chim. Biol..

[54] Haouas M., Taulelle F., Martineau C. (2016). Recent advances in application of Al-27 NMR spectroscopy to materials science. Prog. Nucl. Magn. Reson. Spectrosc..

[55] Gerardin C., Haouas M., Lorentz F., Taulelle F. (2000). NMR quantification in hydrothermal in situ syntheses. Magn. Reson. Chem..

[56] Shi J.M., Anderson M.W., Carr S.W. (1996). Direct observation of zeolite a synthesis by in situ solid-state NMR. Chem. Mater..

[57] Miladinovic Z., Zakrzewska J., Kovacevic B., Bacic G. (2007). Monitoring of crystallization processes during synthesis of zeolite A by in situ (27)A1 NMR spectroscopy. Mater. Chem. Phys..

[58] Ferey G. (1995). Oxyfluorinated microporous compounds ULM-n—Chemical-parameters, structures and proposed mechanism for their molecular tectonics. J. Fluor. Chem..

[59] Ferey G. (2007). The new porous solids: Miracles in the holes. Actual Chim..

[60] Surble S., Millange F., Serre C., Ferey G., Walton R.I. (2006). An EXAFS study of the formation of a nanoporous metal-organic framework: Evidence for the retention of secondary building units during synthesis. Chem. Commun..

[61] Taulelle F., Loiseau T., Maquet J., Livage J., Ferey G. (1993). Oxyfluorinated microporous compounds. 2. Solid-state NMR of (NH_4_)_0.88_(H_3_O)_0.12_AlPO_4_(OH)_0.33_F_0.67_. J. Solid State Chem..

[62] Taulelle F., Pruski M., Amoureux J.P., Lang D., Bailly A., Huguenard C., Haouas M., Gerardin C., Loiseau T., Ferey G. (1999). Isomerization of the prenucleation building unit during crystallization of ALPO_4_-CJ2: An MQMAS, CP-MQMAS, and HETCOR NMR study. J. Am. Chem. Soc..

[63] Goesten M.G., Magusin P., Pidko E.A., Mezari B., Hensen E.J.M., Kapteijn F., Gascon J. (2014). Molecular Promoting of Aluminum Metal-Organic Framework Topology MIL-101 by *N*,*N*-Dimethylformamide. Inorg. Chem..

[64] Haouas M., Volkringer C., Loiseau T., Ferey G., Taulelle F. (2012). In Situ NMR, Ex Situ XRD and SEM Study of the Hydrothermal Crystallization of Nanoporous Aluminum Trimesates MIL-96, MIL-100, and MIL-110. Chem. Mater..

[65] Loiseau T., Lecroq L., Volkringer C., Marrot J., Ferey G., Haouas M., Taulelle F., Bourrelly S., Llewellyn P.L., Latroche M. (2006). MIL-96, a porous aluminum trimesate 3D structure constructed from a hexagonal network of 18-membered rings and mu(3)-oxo-centered trinuclear units. J. Am. Chem. Soc..

[66] Volkringer C., Popov D., Loiseau T., Ferey G., Burghammer M., Riekel C., Haouas M., Taulclle F. (2009). Synthesis, Single-Crystal X-ray Microdiffraction, and NMR Characterizations of the Giant Pore Metal-Organic Framework Aluminum Trimesate MIL-100. Chem. Mater..

[67] Volkringer C., Popov D., Loiseau T., Guillou N., Ferey G., Haouas M., Taulelle F., Mellot-Draznieks C., Burghammer M., Riekel C. (2007). A microdiffraction set-up for nanoporous metal-organic-framework-type solids. Nat. Mater..

[68] Stavitski E., Goesten M., Juan-Alcaniz J., Martinez-Joaristi A., Serra-Crespo P., Petukhov A.V., Gascon J., Kapteijn F. (2011). Kinetic Control of Metal-Organic Framework Crystallization Investigated by Time-Resolved In Situ X-Ray Scattering. Angew. Chem. Int. Ed..

